# Retraction Note: Associations of composite dietary antioxidant index with cardiovascular disease mortality among patients with type 2 diabetes

**DOI:** 10.1186/s13098-025-01595-x

**Published:** 2025-01-17

**Authors:** Chan Yang, Qiangfei Yang, Xi Peng, Xinqiong Li, Guocheng Rao

**Affiliations:** 1 Division of Endocrinology and Metabolism, State Key Laboratory of Biotherapy, West China Hospital, Sichuan University and Collaborative Innovation Center of Biotherapy, Chengdu, 610041 Sichuan China; 2 Jianyang City People’s Hospital, Chengdu, 610040 Sichuan China

Retraction note

**Diabetology & Metabolic Syndrome (2023) 15:131**

10.1186/s13098-023-01109-7

The authors have retracted this article after a concern that it reports NHANES data on manganese, but NHANES does not contain data on manganese. The authors have stated that due to a language barrier manganese was mentioned in the article instead of magnesium. The authors have been offered to submit a revised manuscript for further peer review. Chan Yang has stated on behalf of the authors that they agree to this retraction.

